# Supplementary material for: Speech-in-noise, psychosocial, and heart rate variability outcomes of group singing or audiobook club interventions for older adults with unaddressed hearing loss: A SingWell Project multisite, randomized controlled trial, registered report protocol
Source: PLoS One. 2024 Dec 4;19(12):e0314473. doi: 10.1371/journal.pone.0314473 (PMC11616889; doi:10.1371/journal.pone.0314473)
Supplement: S1 Appendix — (PDF) [file pone.0314473.s001.pdf]

TorontoMet Home > Online Ethics Submission and Review System > Protocol View

Protocol View

APPLICATION CHECKLIST

Protocol Submitted By: chi.lo

Submission Status: Submitted for review

REB: 2024-103

Title of Research Proposal
Benefits of choir for older adults with untreated hearing loss

SECTION 1 - COMMENTS TO CHAIR

Comments to Chair

Please note that this is a multisite study with international partners. However, this REB application is applicable only for our site at TMU. Each other site will be developing their own ethics application. In a discussion with the REB, it was conveyed that commencement of our study at TMU would not require ethics approval from all of the sites involved in the broader multisite study - as this would lead to unnecessary delays, given each site will be operating at a different timeframe. Furthermore, a custom data transfer agreement (DTA) is being developed and is currently being reviewed by OVPRI and Legal. Katherine Spencer and Christopher Hendershot are our contacts for this. A copy of the DTA is attached, noting that it is not finalized but likely indicative. This agreement essentially indicates that we may receive de-identified data from our multisite partners, as we are the lead site developing this international collaborative project.

SECTION 2 - INTRODUCTION & INVESTIGATORS

Principal Investigator

First Name:	Chi Yhun
Last Name:	Lo
Institution:	Toronto Metropolitan University
Academic Title:	Research Associate
Department/Office:	Psychology
Email:	chi.lo@torontomu.ca
Telephone Number:	6477656590
Type:	Other: Research Associate

Investigator Experience

Please provide a brief summary of the investigator's relevant research experience/training (there is no need to include a curriculum vitae). If the principal investigator is a graduate student and the research is being conducted for a thesis or dissertation project, also include a brief summary of the faculty member who is supervising the research.

Dr. Chi Yhun Lo is a Research Associate at TMU, an Honorary Fellow at the Australian Institute of Health Innovation, an Adjunct Fellow at Macquarie University, and Secretary of the Parents of Deaf Children. His work intersects hearing, deafness, music, and health. He has Deaf Awareness Training competencies; and other qualifications relevant to this project include: a Certificate in Australian Sign Language (Auslan) I, and a Certificate in Music for Children with Hearing Loss. He is a Member of the Science of Music Auditory Research and Technology (SMART) Lab, the Alliance for Healthier Communities, and an Affiliate Member of the Music and Health Research Institute. He has provided consulting services with Cochlear Limited (the market leader for cochlear implants) having helped

develop “Bring Back the Beat”, a music-based app designed for cochlear implant users. Finally, Dr. Lo has experience effectively leading multidisciplinary projects having received ~$1,320,000 for research-based projects, and ~$570,000 for community-based projects such as the creation of hearing health workshops for adolescents and young adults; and the creation of STEM workshops for deaf and hard-of-hearing children.

Co-Investigator(s)

First Name: Arla
Last Name: Good
Institution:
Academic Title: Post Doctoral Fellow
Department/Office: Psychology
Email Address: arla.good@torontomu.ca
Telephone Number:

First Name: Frank
Last Name: Russo
Institution:
Academic Title: Professor
Department/Office: Psychology
Email Address: russo@torontomu.ca
Telephone Number:

Student Researcher(s)

No student researchers were specified in this protocol.

Access to Protocol

BB Username	On Email List	Email Address
chi.lo	Y	chi.lo@torontomu.ca
russo		
arla.good	Y	arla.good@torontomu.ca
rhiannon.ueberholz	Y	rhiannon.ueberholz@torontomu.ca
kww	Y	kww@torontomu.ca

SECTION 3 - ABSTRACT

ABSTRACT

Please provide a brief abstract of no longer than 400 words. The abstract **must be in lay terms.** Please avoid jargon and scientific terms. Please include a brief description of the methods, potential benefits, potential risks and risk management procedures.

With a rapidly aging population, age-related hearing loss (or presbycusis) is one of the greatest challenges the world faces. Age-related hearing loss is associated with poor speech perception, social isolation, and loneliness. Furthermore, the majority of adults tend not to address their hearing loss (i.e., untreated hearing loss is more common than hearing aid use). On the other hand, participation in choirs has been shown to support the broadening of social networks and the acquisition of new skills in typical-hearing populations; and the enhancement of speech and pitch perception in hearing aid users. Thus, we hypothesize a choir intervention may be particularly effective at either limiting risk or improving the negative outcomes associated with age-related hearing loss. Our study design is a multisite, randomized controlled trial examining the benefits of choir singing for older adults with untreated hearing loss. The active intervention will received group-based choir training while the control group will be in an audiobook club. TMU is the lead organisation, collaborating with 6 other sites. The outcomes of interesting include: speech-in-noise perception, emotion perception, and psychosocial wellbeing. We will use online questionnaire responses, behavioral responses to listening tasks, and biomarkers such as saliva and heart rate analysis. The potential benefits of the choir intervention include enhanced speech perception, communication, and psychosocial wellbeing. We do not anticipate any risk to participating in this

study, either from the intervention or the test sessions. Our risk management procedure involves informing participants of what participation entails, requiring consent, and that withdrawing from the study can occur at any time and carries no negative consequences.

SECTION 4 - FUNDING

Is the research being funded or is there an application for funding being reviewed currently?

Funded

Sponsor(s)

Name:	The SingWell Project
Cost Centre:	1-51-48151
Funding Period Begin:	July 01, 2023
Funding Period End:	June 30, 2025
Awarded/Requested Amount:	130000.00
Status:	Funded

Please provide additional relevant information regarding the sponsor and the nature of the relationship between the sponsor and the research.

The SingWell Project is a SSHRC-funded project that explores the benefits of choir for a range of populations that have communication disorders. Examples include Parkinsons, Aphasia, and hearing loss.

SECTION 5 - REVIEW BY ADDITIONAL RESEARCH ETHICS BOARDS

a. Does this study involve other institutions or sites or are co-investigators affiliated with other institutions? NOTE: If "yes", your study may require additional REB review at another institution.

Please note that federal guidelines state that REB approval must be provided from ALL relevant REBs as well as Ryerson's REB **before research can begin. This may affect your timelines for starting your research.**

✓ YES

Institution: Memorial University of Newfoundland
Status: Pending

Institution: University Medical Center Groningen
Status: Pending

Institution: Concordia University
Status: Pending

Institution: University of Southern California
Status: Pending

Institution: University of Oldenburg
Status: Pending

Institution: Flinders University
Status: Pending

b. Does this study involve one of both of the following:

- Recruitment of students in elementary and secondary schools (public, private)?
- Recruitment of teaching or administrative staff in elementary or secondary schools (public or private)?

NO

c. Does this study involve one or both of the following:

- A co-investigator whose institutional affiliation is at a hospital or clinic?
- The recruitment of patients, or staff in any hospitals or clinics?

NO

SECTION 6 - OTHER APPROVALS TO CONDUCT RESEARCH

Do you foresee any other research ethics permissions that may be required to conduct this research?

These may include situations such as:

- Formal permission to conduct research in an agency or institution;
- Permission to access documents or personnel in a company;
- Permission from a School Principal to recruit staff, or students in a school.
- Permission from a First Nations, Inuit or Métis community/organization (see Chapter 9 of the Tri-Council Policy Statement) http://www.ethics.gc.ca/eng/policy-politique/initiatives/tcps2-eptc2/chapter9-chapitre9/

NO

SECTION 7 - START AND COMPLETION DATES

Please note that estimated time from a full submission to **first response** from the REB is approximately 4-6 weeks. Time to approval varies with each application, but a first response will be sent to you in 4-6 weeks. The date that you anticipate to begin recruitment should not be a date before that unless you have already contacted the REB Chair or REB Coordinator to discuss this.

Begin Recruitment Date:	01 Jul 2024
Anticipated Completion Date:	30 Jun 2025

SECTION 8 - SECONDARY DATA, HEALTH RECORDS AND CONTENT ANALYSIS

(a) Are you conducting secondary data and/or content analysis of existing data?

NO

(b) Are you Conducting a secondary analysis of biological materials?

NO

(c) Does this study involve the content analysis of existing documents and records that are NOT public records?

NO

(d) Does your research involve the transfer of personal health information and or other data across organizations?

YES

If yes, a data transfer agreement between Toronto Metropolitan University and the originating organization is required. Please attach a copy of the Data Transfer Agreement at the end of this application. If you require additional information regarding data transfer agreements please, contact the OVPRI or a REB Coordinator.

SECTION 9 - SCHOLARLY REVIEW

The research protocol has undergone scholarly review prior to this submission for ethical review, e.g. has been reviewed by a granting agency, thesis/MRP committee or supervisor or other review committee.

Please provide details:

This methods protocol has undergone a review process with the SingWell project Committee and underwent review by the grant agency. Furthermore, we intend to register the study's methods protocol. Hence, it will undergo an additional peer review after the study has obtained TMU REB ethics clearance.

SECTION 10 - CONFLICT OF INTEREST

Are there any potential or perceived conflicts of interest that you foresee in conducting the research?

NO

SECTION 11 - RESEARCH METHODS / DESIGN

a. Please check all research designs and/or methods that apply to your project:

Quantitative Research
Internet Based or Online Research. [please complete Internet-Based or Online Research section]

b. Please check all methods of collecting data that may apply:

In-person interviews
  Maximum number of participants for this method: 30
Phone or Skype interviews
  Maximum number of participants for this method: 30
Surveys or questionnaires
  Maximum number of participants for this method: 30
Audiorecording
Videorecording
Taking photographs
Collection of biological material
  Maximum number of participants for this method: 30

c. Please include a brief review of literature. The review of literature should provide background literature that substantiates your research approach/design/questions. The review of literature should include similar studies/projects that have been conducted and have lead to the rationale for your research and your project design. The review of literature might also include applicable literature about your theoretical framework. Please avoid discipline specific jargon. You may include your reference list as an attachment in the final section but the review of literature must be in the text box below.

Age-related hearing loss (or presbycusis) is one of the greatest challenges the world faces, given a rapidly aging population. Age-related hearing loss is associated with poor speech perception, social isolation, loneliness, and cognitive decline (Shukla et al., 2020). On the other hand, there is evidence that music-training enhances listening abilities, with two systematic reviews finding evidence for a "musician advantage", particularly with speech-in-noise perception. Another recent review suggests that one of the most important benefits are the social-emotional benefits of music, particularly in group contexts (Schellenberg & Lima, 2024). Furthermore, participation in choirs has been shown to support the broadening of social networks and the acquisition of new skills in normal-hearing populations (Sole et al., 2010); and the enhancement of speech and pitch perception in hearing aid users (Dubinsky et al., 2019). Therefore, while there is some growing evidence that choir may be beneficial for older adults with hearing loss, the literature supporting

these findings remains small. Thus, we aim to contribute to this area of investigation, hypothesizing that choir-based interventions may be particularly effective at either limiting risk or improving the negative outcomes associated with age-related hearing loss. Reference List: Dubinsky, E., Wood, E. A., Nespoli, G., & Russo, F. A. (2019). Short-Term Choir Singing Supports Speech-in-Noise Perception and Neural Pitch Strength in Older Adults With Age-Related Hearing Loss. Frontiers in Neuroscience, 13. https://doi.org/10.3389/fnins.2019.01153 McKay, C. M. (2021). No Evidence That Music Training Benefits Speech Perception in Hearing-Impaired Listeners: A Systematic Review. Trends in Hearing, 25. https://doi.org/10.1177/2331216520985678 Schellenberg, E. G., & Lima, C. F. (2024). Music training and nonmusical abilities. Annual Review of Psychology, 75, 87-128. Shukla, A., Harper, M., Pedersen, E., Goman, A., Suen, J. J., Price, C., Applebaum, J., Hoyer, M., Lin, F. R., & Reed, N. S. (2020). Hearing Loss, Loneliness, and Social Isolation: A Systematic Review. In Otolaryngology - Head and Neck Surgery (United States) (Vol. 162, Issue 5, pp. 622–633). SAGE Publications Inc. https://doi.org/10.1177/0194599820910377 Sole, C., Mercadai-Brotons, M., Gallego, S., & Riera, M. (2010). Contributions of Music to Aging Adults' Quality of Life. In Journal of Music Therapy: Vol. XLVII (Issue 3). https://academic.oup.com/jmt/article/47/3/264/862365

d. Please provide information regarding the design of the proposed research and data collection. Specify aims of the research that may include the expected outcomes or goals of the research, any hypotheses to be tested, or research questions to be answered. Please also include the timelines for your research project.

The design of the proposed research is a randomized controlled trial. The aim is to explore the benefits of choir participation for adults 60 years and older with untreated hearing loss (i.e., adults with hearing loss that do not use a hearing aid or cochlear implant). The control group will consist of an 'Audiobook Club' which will match the choir group for expectation, social and communication interaction, and duration of training. Audiobooks are a suitable control as they a common recommendation for adults that have recently received as hearing aid or cochlear implant. The recruitment target is 30 participants (15 for choir training, 15 for audiobook club). To reduce bias, experimenters will be blinded with respect to participant experimental group. We hypothesize that choir-based training choir will generate specific benefits for perceptual skills such as the enhancement of pitch, rhythm, and timbre. These perceptual measures are associated with speech-in-noise perception and understanding emotional prosody. We also anticipate group-based choir activities will improve social connections and bonds due to the synchronous and collective goal-oriented nature of choir singing, as well as decrease stress. TRAINING: The choir and audiobook training will occur over a 12-week period and will be videorecorded. Participants will be randomly selected to either the choir or audiobook group. Choir participants will attend a 1.5 hour choir singing session (facilitated by a choir instructor), once-a-week over 12-weeks. Audiobook participants will attend a 1.5 hour audiobook club session (with a facilitator), once-a-week over 12-weeks. Participants will listen to an audiobook chapter prior to each session, and will collectively discuss this each week. All sessions will also be attended by a Research Assistant level volunteer to help the facilitator, as well as encourage the participants to sing or share their thoughts on the audiobook. SCREENING: Screening will occur at the first test-session. All participants will undergo a short audiometric screener to confirm their hearing levels. All participants will be screened for cognition using the MoCA-HI (Montreal Cognitive Assessment for Hearing Impaired). TESTING: The test sessions will occur over a 24-week period, stratified as MACRO and MICRO timepoints. MACRO timepoints: participants will be tested at PRE (Week 0, to measure baseline abilities), POST (Week 13, to measure any change to baseline abilities), and FOLLOW-UP (Week 24, to measure retention of any benefit after training has ceased). Each test session is anticipated to take 1-hour to complete. Participants will also complete online questionnaires prior to each test session. Behavioral test measures include: 1) speech-in-noise test, 2) emotional speech test, and 3) music/auditory tests. Participants will listen to 1) a sentence in the presence of noise and click on the selection they heard; 2) a sentence spoken in an emotional state that the participants must identify; 3) music perception tests related to identifying differences in pitch, rhythm, and timbre. MICRO timepoints: participants will be tested before and after the training at Weeks 2, 7, and 11 with the collection of saliva and heart rate variability via a 'Polar Verity Sense' an optical heart rate sensor (to quantify levels of stress). A full description of all measures such as questionnaires and listening tasks is attached. Note, these are all validated and have been used in SingWell studies. Statistical design will be targeting comparisons between test and control training outcomes—across groups statistical effects. For example, a significant Group effect (choir, control), Session (PRE, POST, FOLLOW-UP) interaction will indicate training differs in the two groups. Post-hoc statistical tests will indicate potential mechanisms for benefit. The study is anticipated to begin in 2024, with completion in 2025.

e. Will you be collecting demographic data from participants?

YES

If YES, please indicate what data will be collected (and attach any demographic data collection forms to the protocol):

Age
Gender/Sex
Address
E-mail address

If you are collecting and storing: NAME, ADDRESS, TELEPHONE NUMBER and/or E-MAIL ADDRESS, please provide your reasons for collecting and storing this personal and identifiable data, and explain how it will be used:

As we are the lead site for a multisite study across a number of cities/countries around the world, each site will code their participants by country (i.e., Canada, USA, Amsterdam, Germany, Australia) to allow for country-based comparisons. Thus, we are not collecting a full address, simply labelling our participants by their country of participation. Email addresses will be required so that we can contact participants throughout the study. For example, to send out online questionnaires, to organise their intervention (choir or audiobook club) sessions and the testing sessions. Email addresses will not be stored in the same file as participant results. Finally, all data will be secured on password protected computers, with backups on Google Drive and the SMART Lab's network drive. Two-factor authentication (with the Google authenticator app) is also required to access these files.

f. If you will be collecting demographic data on ethnicity, will this variable include 'Aboriginal' (First Nations, Inuit or Métis)?

NO

SECTION 12 - CLINICAL TRIAL OR INTERVENTION RESEARCH

1. Does this research evaluate the effects of one or more health-related interventions or health outcomes? Interventions include, but are not restricted to, psychotherapies, drugs, radiopharmaceuticals, cells and other biological products, surgical procedures, radiologic procedures, devices, genetic therapies, natural health products, process-of-care changes, preventive care, and manual therapies. Clinical trials may also include questions that are not directly related to therapeutic goals – for example, drug metabolism – in addition to those that directly evaluate the treatment of participants.

NO

SECTION 13 - SELECTION AND RECRUITMENT

Recruitment Guidelines

a. Inclusion and Exclusion criteria

Please describe, in detail, the inclusion and exclusion criteria for potential participants.

Inclusion criteria - Adults aged 60 years and older. - Untreated hearing loss (i.e., not using assistive listening device such as a hearing aid or cochlear implant). - Sufficient English ability to understand the questionnaire and test materials. - Have neurotypical cognition for their age, measured using the Montreal Cognitive Assessment - Hearing Impaired (MoCA-HI). Exclusion criteria: - Uses a hearing aid or assistive listening device.

b. How will recruitment be conducted? Please describe **all possible** ways that you are planning on recruiting potential participants. If you will be emailing or phoning, please include how you will access contact information such as email addresses and phone numbers.

Older participants will be drawn from a hearing database maintained by the SMART lab, which currently includes over 400 older adults with varying levels of hearing ability. Participants fitting our criteria will be contacted via email using this script (please note, for the purposes of not biasing our participants or setting up expectations; we will list the study title as "Exploring the benefits of creative group activity" on the recruitment materials.): Hello, My name is Dr. Chi Yhun Lo. I am a Research Associate at Toronto Metropolitan University in the SMART Lab. I am contacting you to see if you might be interested in participating in a research study. The focus of the research is to

explore the benefits of creative group activity. To participate you need to be aged 60 years and older, be fluent in English, and not use a hearing aid or other assistive listening device. If you agree to volunteer, you will be randomly assigned to join a creative activity such as a choir or an audiobook club. We anticipate these activities may help you learn new skills and provide opportunities to meet peers and socialize. You will complete questionnaires, be tested on listening abilities, and we will monitor stress levels through the collection of saliva samples and heart rate. We can assure you this is a very simple, non-invasive task. Your participation in the group-based creative activities will occur over a 12-week period, requiring a commitment of 1.5 hours, once-a-week. Three testing sessions will occur: 1) the week before the activity starts; 2) the week after the activity finishes; and 3) 12-weeks after the activity has finished. In appreciation of your time, you will receive $20 per test session. Your participation is completely voluntary and if you choose not to participate it will not impact your relationship with the SMART Lab or Toronto Metropolitan University. The research is funded by the SingWell Project and has been reviewed and approved by the Toronto Metropolitan University Research Ethics Board. If you are interested in more information about the study or would like to volunteer, please reply email Dr. Chi Yhun Lo (chi.lo@torontomu.ca).

c. Are there any already-existing relationships between the researcher and potential participants that may possibly contribute to feelings of obligation or undue influence to take part? (e.g. instructor-student, service-provider-client, manager-employee, etc.)

NO

If YES, please describe these already-existing relationships and outline strategies you will put in place to avoid potential participants feeling unduly obligated to take part, e.g. having someone other than the researcher inform the potential participants about the study):

d. Will you be using recruitment flyers/brochures/advertisements to recruit potential participants?

NO

e. Will you be sending e-mails or using social media to recruit participants (e.g. Twitter, Facebook)?

YES

If YES, please attach e-mail recruitment notice, 140-character tweet, or FB notice.

f. Will you be using in-person or phone recruitment?

NO

g. Will you be recruiting from introductory psychology courses at Toronto Metropolitan University (PSY 102 or PSY 202)?

NO

h. Will you be recruiting from the Ted Rogers School of Management Student Research Pool?

NO

SECTION 14 - CONSENT

Consent/Assent Form Instructions
Online Consent Survey

a. Will you be obtaining consent?

YES

If YES, Please describe the process by which consent will be obtained. Please describe when the consent process will occur, who will obtain informed consent and confirm that there is adequate time for discussion and questions:

Consent will be obtained before commencement in this study. If participants indicate their willingness to participate in our study (via the email invitation), participants will be emailed the Consent Form via Qualtrics. They will be prompted to read through the interview consent form and encouraged to respond with any questions before submitting their response. There is no time pressure, and participants have as long as they would like to agree to participate (or not) in our study. There is no penalty to not participating or withdrawing from the study at any time.

b. What method of obtaining consent are you using?

Consent form before an on-line survey (attach consent form at end of application)

c. Are you providing the potential participants with the consent form ahead of the time of participation in order for them to have adequate time to reflect upon potential participation? (If not applicable, simply indicate N/A)

YES

If YES, please indicate how you will be providing the consent form to participants before their participation:

As previously indicated, participants will be drawn from a hearing database maintained by the SMART lab and sent an email invitation for the proposed study. Hence, they will have adequate time to reflect upon their participation in their own time, without any external influence.

d. Does your research involve any persons who cannot provide consent for themselves and require a guardian, proxy or substitute decision-maker to provide proxy consent?

NO

SECTION 15 - VOLUNTARY NATURE OF PARTICIPATION

a. Please describe strategies you will use to ensure participants know that they have the right to withdraw voluntarily from participation or parts of participation. Please describe how participants will be informed about this right in the informed consent process. Please also indicate what will happen to their data if they withdraw after or during participation:

On the consent page, we will emphasize that participants can withdraw at any point, and their data will not be utilized and deleted immediately. Similarly, if at any point during the study, if participants are distressed, the training or testing will be paused immediately, and the participant will be asked if they are okay or would like further support. They will be able to withdraw entirely from the study, and they will be reminded that their participation is completely voluntary and they have the right to withdraw at any time in the future. If they are willing to continue, they may do so. Please note: we are not anticipating any distress - this is based on our previous experience running choir-based studies in the past. Nonetheless, we are prepared for this possibility.

SECTION 16 - POTENTIAL RISK AND BENEFIT

a. What level of potential risk does this research involve, for participants?

Low/minimal risk

b. Please identify the kinds of potential risks and briefly describe the risk and why you feel the risk is minimal, medium or high:

- **Social risk (e.g. being exposed or embarrassed, potential loss of privacy, damage to reputation)**
  Describe the risk:

  While it is not anticipated that being in a choir or audiobook club will cause distress, we are aware of these possible risks, and strategies are in place to mitigate any stress as a result of the questions presented. The audiobook club facilitator will also set clear ground rules to ensure the discussion is respectful. Other potential risks could include being insecure in singing, or being shy to share their ideas on the audiobook.

  How will the risk be managed minimized or mitigated?

  Both the choir and audiobook clubs will be facilitated and moderated by an individual that has experience managing a choir or bookclub. Additionally, the presence of a Research Assistant level volunteer is designed to encourage participants to either share their singing or thoughts on audiobooks. Finally, the group-based nature of the training means that there is not necessarily pressure on any one participant to perform or share. For example, a participant in the choir group will always be singing as a group (i.e., to some extend a participant can 'hide' their voice within the group); or if a participant does not want to share their thoughts on an audiobook, that is fine - the facilitator can easily move onto someone else or another discussion point.

c. Does this research involve any potential **group risks**.
This could include potential risks to groups of persons identified or under study. (e.g. risk of being further marginalized as a result of the research, risk of being denied access to services as a result of the research, risk of being identified as a group of individuals within an organization, etc.)

NO

d. The Tri-Council Policy states that vulnerability is often caused by limited access to social goods, such as rights, opportunities and power. Individuals or groups in vulnerable circumstances have historically included children, the elderly, women, prisoners, those with mental health issues and those with diminished capacity for self-determination. (See page 10 of Tri-Council Policy Statement 2 http://www.pre.ethics.gc.ca/eng/policy-politique/initiatives/tcps2-eptc2/Default/ Article 4.7 of the TCPS2 states that individuals and groups in vulnerable circumstances should not be inappropriately or automatically excluded.
Does this research involve individuals or groups whose circumstances may make them vulnerable in the context of the research?

NO

e. Please describe potential benefits from the research to participants and, including benefits to individuals, groups, communities and societal benefits. Benefits do not include incentives, reimbursement or compensation for participants. Please see section on Incentives, Reimbursement and Compensation.

Participants may improve their speech perception ability, develop social bonds (and reduce social isolation), as well as reduce stress. Participants may also derive a sense of benefit by contributing to our understanding of group-based creative activities. We cannot guarantee any direct benefit from participation in the proposed study.

SECTION 17 - PRIVACY AND CONFIDENTIALITY

Confidentiality - means that the information shared and all data collected will be kept secret and not shared. Although you may meet with research participants or you may have data that could be used to identify participants, this information will be kept confidential. No identifying information will be included in the dissemination of the results.

Anonymity - means that at no time will the researcher or anyone associated with the research know of the identity of participants. The term anonymous may be used in conjunction with surveys that are

completed and submitted without any identifying information included.

For more information on Privacy and Confidentiality please see the Tri-Council Policy Statement chapter on Privacy and Confidentiality at http://www.pre.ethics.gc.ca/eng/policy-politique/initiatives/tcps2-eptc2/chapter5-chapitre5/

Based on the definitions above, please specify whether participants will be anonymous to the researcher or, if known to the researcher, please describe what measures will be undertaken to ensure their identity will remain confidential. If the research design involves both anonymity and confidentiality, please provide details below.

The participants will be anonymous to the research team, we will not be linking the database we are recruiting the participants from to our dataset. All information and data will remain confidential and anonymous. Email addresses will be kept separate from the dataset and will be stored on a secure password-access Toronto Metropolitan University-based server (SMART Lab Google Drive).

If participant anonymity or confidentiality is not relevant or appropriate to this project, please explain:

State who will have access to research data:

Members of this research team (Chi Yhun Lo, Arla Good, Frank Russo, Rhiannon Ueberholz, and Kay Wright-Whyte.

SECTION 18 - DATA STORAGE

Please describe what data will be collected (i.e. datasets stored electronically , signed consent forms, audio or video files, transcripts, etc.). For more information on Privacy and Confidentiality please see the Tri-Council Policy Statement chapter on Privacy and Confidentiality at http://www.pre.ethics.gc.ca/eng/policy-politique/initiatives/tcps2-eptc2/chapter5-chapitre5/

Online consent forms and questionnaire will be collected via Qualtrics. Consent form data, Qualtrics questionnaire data, behavioral test data (e.g., speech perception scores, music perception scores), audio and video recordings, data from salivary analysis, and heart rate data will be stored on password-protected computers in the SMART Lab at TMU, which will be backed up on Google Drive and the SMART Lab password-protected network drive. Data will be labelled using unique numerical labelling that is stored separately from identifying documents (i.e., consent forms) and as password-protected folders to which Chi Yhun Lo, Arla, Good, Frank Russo, Rhiannon Ueberholz, and Kay Wright-Whyte.

If data will be transported (e.g. shared between research partners, etc.) describe the process in which data will be securely transported.

If research partners request the data for purposes of verification of the results, the transfer will be secure and electronic. No individual identifiers will be provided with the data (all confidential information, names and emails, will be kept separate from the anonymous data). Electronic transfer will be encrypted and occur through Google Drive (using a TMU account).

Please note how long data will be kept for and the rationale for keeping the data for this length of time. Also indicate how data will be destroyed after this time.

All data are necessarily de-identified and retained indefinitely for future analyses. Access to the data will only be granted to authorized members of the SMART Lab. It is possible that a third party (e.g., graduate students who may become involved the research at a future date) will have access to the data for a purpose that was not originally identified in this study. As well, results may be shared with others at scholarly meetings or as part of published papers. However, all information will be presented so that no individual information will be identifiable in any way. De-identified data may be

provided to other researchers for the purposes of the study or verification of results; any data that is shared will NOT include the names of ANY participants.

SECTION 19 - DATA DISSEMINATION

Please describe how data will be disseminated (e.g. conference presentations, journal publications, book, etc.):

Findings from this study will be disseminated to the community by way of conference presentations and peer-reviewed publications. All findings will involve de-identified datasets.

Please indicate if and how participants will be provided with a copy of the research findings. If so, please also ensure that this is indicated on the consent form.

At their own leisure, participants will be able to access research findings posted on the Science of Music, Auditory Research and Technology (SMART) Lab website. Participants may choose to request their individual data and can email the research team directly (email details will be provided on the initial email invitation to participate, and consent form).

SECTION 20 - INCENTIVES, REIMBURSEMENTS AND COMPENSATION

Please read the Guideline on Incentives, Reimbursement and Compensation provided by the REB prior to completing this section.
http://torontomu.ca/content/dam/research/documents/ethics/REB Guidelines for Incentives Reimbursements and Compensation for Research Participants.docx

Will you be providing incentives to participants?

YES

If YES, please describe the compensation being provided (financial, bonus credit, entry into a draw):

Participants will be incentivized to participate in the study to learn new skills (i.e., singing or learning through audiobooks), and through opportunities to socialize with peers. Participants will also be reimbursed $20 for each test session (3 test sessions in total).

Will you be reimbursing participants for out of pocket expenses?

NO

Is there a substantial risk of physical injury for participants?

NO

SECTION 21 - STUDY LOCATION(S)

PLEASE READ THE FOLLOWING NOTE
Interviews should be conducted in a location that provides aural and visual privacy. If public areas such as coffee shops will be used for interviews please provide a rationale for such location and indicate any potential challenges to visual privacy and how you will ensure aural privacy. Please also note that participants should ideally not be interviewed in their workplace unless it offers aural and visual privacy. For reasons of personal safety research should not be conducted at the home of the participant or the researcher.

a. Please describe where the research will take place.

Training and testing will occur on TMU campus, in spaces located in South Bond Building (SBB) and Atrium on Bay (AOB).

SECTION 22 - INTERNET-BASED RESEARCH

Are you conducting Internet-based research?

YES

(A) If YES, please describe the type of internet research (e.g. collecting information from private chat rooms, conducting an on-line survey – FluidSurvey, SurveyMonkey or Opinio).

We will be collecting questionnaire based data using Qualtrics.

(B) Will you be administering an on-line survey?

YES

If YES, participants must still be able to provide full and informed consent. Typically, this is done by a consent preamble, followed with a "button" to note agreement that takes the participant to the survey. This consent preamble must include all of the necessary elements of an informed consent process. [link to researcher guideline on consent]

(C) Have you provided a consent preamble for participants that will be attached to the survey?

YES

IF YES, please attach at the end of the application.

(D) If participants discontinue participation part way through the survey or online activity will the data completed up to that point be collected and analyzed.

NO

(E) Have you indicated, in the consent preamble, how participants can discontinue participation if they wish (i.e. "simply close your browser and no data will be collected")

YES

(F) Do you have specific inclusion or exclusion criteria (e.g. participants must be a particular age, ethnic group, occupation or gender). If so, please describe how you will ensure that these criteria are met, by on-line participants.

Yes. A series of criteria-based checkboxes will be presented prior to the participant providing consent on the online consent form. The criteria will be verified as part of our screening process during the first in-person test session.

SECTION 23 - INVOLVES INDIGENOUS PEOPLE

[Guidelines for research involving indigenous peoples in canada.pdf]

Are you conducting research that involves First Nations, Inuit or Métis persons? Specifically, does this research involve any of the following:

- research conducted on First Nations, Inuit or Métis lands;
- recruitment criteria that include Aboriginal identity as a factor for the entire study or for a subgroup in the study;
- research that seeks input from participants regarding Aboriginalr cultural heritage, artefacts, traditional knowledge or unique characteristics;
- research in which Aboriginal identity or membership in an Aboriginal community is used as a variable for the purpose of analysis of the research data; or
- interpretation of research results that will refer to Aboriginal communities, peoples, language, history or culture.

NO

SECTION 24 - INVOLVES CHILDREN/YOUTH

Are you conducting research involving children and/or youth?

NO

SECTION 25 - INVOLVES DECEPTION

Are you conducting research involving deception or incomplete disclosure?

NO

SECTION 26 - INVOLVES HUMAN BIOLOGICAL MATERIALS

1. Are you conducting research involving human biological materials?

According to the TCPS, human biological materials includes: human tissues, organs, blood, plasma, skin serum, DNA, RNA, protein, cells, hair, nail clippings, urine, saliva, and other human bodily fluids. It also includes materials related to human reproduction, including embryos, fetuses, fetal tissues, and human reproductive materials.

YES

2. Please clarify the type and amount of biological materials to be obtained/used

Saliva samples (2 mL) over 3 sessions for a total of 6 mL.

3. State the manner in which biological materials will be obtained/used, and the safety and invasiveness of the procedures for acquisition. **If obtained by a third party, such as the ATCC or Cedarlane Corporation, please be sure to upload the Material Transfer Agreement (MTA).**

Saliva samples will be collected using the passive drool method, which entails gathering saliva in the mouth for approximately 1-2 minutes (depending on salivary flow) and spitting into a 2mL polypropylene cryovial (i.e., tube) using a collection aid (supplied by Salimetrics). This is a hygienic non-invasive sampling method that allows participants to easily sample their own saliva without professional assistance. There are no adverse effects associated with this non-invasive collection method. This method has been used in older adult populations. It takes approximately 1-2 minutes to generate enough saliva for each collection tube. All samples will be labeled with a unique study ID and will not be linked to participant identifying information. Once saliva is sampled, tubes will be

stored in a -80 degree C freezer until subsequent assays (an enzyme-based analysis) are performed at TMU.

4. Please state the intended use(s) of the biological materials, including any commercial use.

The intended use of the salivary analysis is to identify changes to biomarkers that are associated with social bonding and stress. There is no commercial use or purpose.

5. Please state the measures employed to protect the privacy of and minimize risks to participants/donors, etc.

All samples will be labeled with a unique study ID and will not be linked to participant identifying information.

6. Please state the length of time the biological materials will be kept, how they will be preserved, location of storage (e.g., in Canada, outside Canada), and process for disposal, if applicable.

Surplus saliva will be stored at TMU for up to 7 years following study completion, which is an estimation of how long samples will remain viable. Following this 7-year storage period, all samples will be securely and safely destroyed according to standard safety protocol of TMU.

7. Please state any anticipated linkage of biological materials with information about the participant.

All samples will be labeled with a unique study ID and will not be linked to participant identifying information. The data will be linked to a de-identified dataset. We will also be collecting information on food/drink participants consumed and any exercise - this questionnaire has been attached.

8. Please state the plan for handling results and findings, including clinically relevant information and incidental findings.

Salivary cortisol and oxytocin are not diagnostic measures, and therefore no clinically relevant information or incidental findings are anticipated. All data will be analyzed as part of the de-identified dataset, which will also be presented as aggregated data.

9. As per Article 12.5 of the Tri-Council Policy Statement, institutions and researchers shall ensure they have or use appropriate facilities, equipment, policies and procedures to store human biological materials safely, and in accordance with applicable standards; and, shall establish appropriate physical, administrative and technical safeguards to protect human biological materials and any information about participants from unauthorized handling.

- [x] I confirm that the research project proposed herein is in adherence with the above statement

10. Does this research involve biological materials related to human reproduction (embryos, fetuses, fetal tissue, etc.)?

NO

If yes, your protocol will be reviewed based on the Assisted Human Reproduction Act. Please see http://laws-lois.justice.gc.ca/eng/acts/a-13.4/

SECTION - ATTACHMENTS

Please attach all research documents here. Please attach only documents pertaining to the human participant section of your research. Please **DO NOT** attach funding proposals, thesis proposals or dissertation proposals.

Please label the attachments with an appropriate descriptive name and version or date. Remember, these may include:

a. Consent form(s) (Please see our instructions for consent/assent forms); http://torontomu.ca/content/dam/research/documents/ethics/REB Consent Assent Form Template.doc
b. Online Consent Survey
c. Recruitment notices, flyers, ads or e-mails;
d. Recruitment Guidelines
e. Instruments, interview guides or surveys;
f. Data collection forms;
g. Applications or approvals from other REBs;
h. Letters or notes of support.
i. Data sharing and/or data transfer agreements.
j. Research agreements with First Nations, Inuit or Métis communities/organizations. (See Tri-Council Policy Statement Article 9.11) www.pre.ethics.gc.ca/eng/policy-politique/initiatives/tcps2-eptc2/Default/
k. Research design chart/s
l. Clinical trial or Intervention details

Uploaded supporting document(s)

- (i) Data Transfer Agreement.pdf (submitted on: 18 Feb 2024)
- (c) Recruitment Email.docx (submitted on: 18 Feb 2024)
- Polar Verity Sense User Guide.pdf (submitted on: 20 Feb 2024)
- (b) Online Consent.docx (submitted on: 20 Feb 2024)
- (e) All questionnaires.pdf (submitted on: 20 Feb 2024)

Print Submission | Export to PDF | Close Window
