# Supplementary material for: Speech-in-noise, psychosocial, and heart rate variability outcomes of group singing or audiobook club interventions for older adults with unaddressed hearing loss: A SingWell Project multisite, randomized controlled trial, registered report protocol
Source: PLoS One. 2024 Dec 4;19(12):e0314473. doi: 10.1371/journal.pone.0314473 (PMC11616889; doi:10.1371/journal.pone.0314473)
Supplement: Attachment — Submitted filename: renamed_45b86.docx [file pone.0314473.s003.docx]

# COMMENTS – Manuscript PONE-D-24-30955

Title: “Speech-in-noise, psychosocial, and heart rate variability outcomes of group singing or audiobook club interventions for older adults with unaddressed hearing loss: a SingWell Project multisite, randomized controlled trial, registered report protocol”

**Important note: This review pertains only to ‘statistical aspects’ of the study and so ‘clinical aspects’ [like medical importance, relevance of the study, ‘clinical significance and** **implication(s)’ of the whole study, etc.] are to be evaluated [should be assessed] separately/independently. Further please note that any ‘statistical review’ is generally done under the assumption that study specific methodological [as well as execution] issues are perfectly taken care of by the investigator(s). This review is not an exception to that and so does not cover clinical aspects {however, seldom comments are made only if those issues are intimately / scientifically related & intermingle with ‘statistical aspects’ of the study}. Agreed that** **‘statistical methods’ are used as just tools here, however, they are vital part of methodology [and so should be given due importance]. I look at the manuscript in/with statistical view point, other reviewer(s) look(s) at it with different angle so that in totality the review is very comprehensive. However, there should be efforts from authors side to improve (may be by taking clues from reviewer’s comments). Therefore, please do not limit the revision only (with respect) to comments made here.**

**COMMENTS:** Because the study & manuscript is excellent in all aspects, there is hardly anything to point-out. However, I have different opinion regarding a few (only two) issues which are given below:

When lines 599-602 it is stated that “Given the sample size and random assignment to group, we assume that covariates such as age, gender, education, musical training/background, credibility, and expectation will be evenly distributed across the participants. Thus, we expect equivalence between participants and their randomly assigned intervention” then why further said “To test this assumption, we will use a well-known technique to test for equivalence, the Bayesian *t*-test”? Test proposed is appropriate and reference quoted [Stefan AM, Gronau QF, Schönbrodt FD, Wagenmakers EJ. A tutorial on Bayes Factor Design Analysis using an informed prior. Behav Res Methods. 2019;51: 1042–1058] is nice. However, it is often very rightly advised that when random allocation/assignment is used/done any statistical comparison of baseline characteristics is not necessary. In this context, I request authors to note that even if P-value(s) turn(s) out to be significant (while comparing baseline characteristics despite random allocation), it is, by definition, a false positive as you then are supposed to be testing ‘randomization’ then, which in any single trial may not balance all baseline characteristics {though I am sure that these learned authors already know these things}.

Please note that any regression techniques are not basically/originally developed for any sort of [between or within group(s)] comparison(s). Using mixed-model regression is very appropriate in given situation, but note that ‘Head-to-head’ comparison is expected, as this is an indirect/secondary/by-product testing, in my opinion.

As pointed out in ‘important note’ above “This review pertains only to ‘statistical aspects’ of the study and so ‘clinical aspects’ should be assessed separately/independently. In my opinion, to make this article acceptable (which is quite possible and easy), a small amount of re-vision (re-drafting) may be needed. ‘Minor revision’ is recommended.
